# Subnormothermic *ex vivo* lung perfusion attenuates ischemia reperfusion injury from donation after circulatory death donors

**DOI:** 10.1371/journal.pone.0255155

**Published:** 2021-08-02

**Authors:** Stephan Arni, Tatsuo Maeyashiki, Isabelle Opitz, Ilhan Inci

**Affiliations:** Department of Thoracic Surgery, University Hospital Zurich, Zurich, Switzerland; Virginia Commonwealth University, UNITED STATES

## Abstract

Use of normothermic *ex vivo* lung perfusion (EVLP) was adopted in clinical practice to assess the quality of marginal donor lungs. Subnormothermic perfusion temperatures are in use among other solid organs to improve biochemical, clinical and immunological parameters. In a rat EVLP model of donation after circulatory death (DCD) lung donors, we tested the effect of four subnormothermic EVLP temperatures that could further improve organ preservation. Warm ischemic time was of 2 hours. EVLP time was of 4 hours. Lung physiological data were recorded and metabolic parameters were assessed. Lung oxygenation at 21°C and 24°C were significantly improved whereas pulmonary vascular resistance and edema formation at 21°C EVLP were significantly worsened when compared to 37°C EVLP. The perfusate concentrations of potassium ions and lactate exiting the lungs with 28°C EVLP were significantly lower whereas sodium and chlorine ions with 32°C EVLP were significantly higher when compared to 37°C EVLP. Also compared to 37°C EVLP, the pro-inflammatory chemokines MIP2, MIP-1α, GRO-α, the cytokine IL-6 were significantly lower with 21°C, 24°C and 28°C EVLP, the IL-18 was significantly lower but only with 21°C EVLP and IL-1β was significantly lower at 21°C and 24°C EVLP. Compared to the 37°C EVLP, the lung tissue ATP content after 21°C, 24°C and 28°C EVLP were significantly higher, the carbonylated protein content after 28°C EVLP was significantly lower and we measured significantly higher myeloperoxidase activities in lung tissues with 21°C, 24°C and 32°C. The 28°C EVLP demonstrated acceptable physiological variables, significantly higher lung tissue ATP content and decreased tissue carbonylated proteins with reduced release of pro-inflammatory cytokines. In conclusion, the 28°C EVLP is a non inferior setting in comparison to the clinically approved 37°C EVLP and significantly improve biochemical, clinical and immunological parameters and may reduce I/R injuries of DCD lung donors.

## Introduction

Lung transplantation is the accepted final treatment option for end stage lung diseases, once all possible conservative treatments have been exhausted. With more than three decades of success stories in the field, the waiting list mortality is still an issue among lung transplant candidates [1]. Novel strategies are implemented to overcome this shortage, such as living donor lobar lung transplantation, application of extended criteria (marginal) donor lungs, donation after circulatory death (DCD) donors [2]. However, in the modern era and even with an increasing number of lung transplantation being performed around the world, the demand for donor lungs is still outpacing scarcity of suitable lung donor organ [1].

*Ex vivo* lung perfusion (EVLP) was developed to increase lung utilization by re-evaluating, treating, and repairing questionable donor lungs before transplantation [3]. Normothermic EVLP is adopted in clinical practice to assess the quality of marginal donor lungs [4]. However, future protocols will require advances to perform longer machine perfusion time for the purpose of efficiently repair questionable lungs [5]. Decreasing the *ex vivo* perfusate temperature has been already used in clinical practice in other solid organs such as liver [6–10] and kidney [11] and has been shown to reduce tissue metabolism and effectively protect from ischemia-reperfusion (I/R) injury [7, 11, 12]. Nevertheless, few investigators have studied which perfusion temperature might be useful during EVLP [12–15].

The main goal of normothermic machine perfusion is to optimize graft preservation by mimicking physiological conditions and allows the continuous elimination of toxic products from the cellular milieu and actively restores ATP and glycogen reserves. Normothermic perfusion enables organ viability assessment before transplantation, prolonged preservation, and resuscitation from injuries. In this study, we investigated the effect of perfusion temperature as one of the key factor for graft improvement. Nonetheless, it is still unknown which condition is physiologically advantageous for DCD lung organ preservation among hypothermic lung perfusion (4–10°C, [12]), subnormothermic lung perfusion (20–33°C [13]), or normothermic lung perfusion (37°C). In this study, we demonstrated the physiological as well as biochemical, clinical and immunological parameter improvements to clarify the cytoprotective benefits of reduced cellular metabolism under subnormothermic lung perfusion temperatures that could further improve organ preservation in DCD lung donors.

## Materials and methods

### Animals

Protocols of animal experiments for this study were approved by the Veterinary Office of the Kanton Zürich, Switzerland (reference ZH228/16). Pathogen-free male Sprague Dawley rats (Janvier Labs, France) of 280–360g weight were bred in specific pathogen-free area and treated according to the “Guide for the Care and Use of Laboratory Animals: Eighth Edition” [16].

### Surgical techniques for donation after circulatory death donor lung and EVLP model

The rats were anesthetized with isoflurane and underwent tracheotomy and mechanical ventilation with a rodent ventilator (Harvard Apparatus, Inc., Model Ventelite, Germany). We selected a tidal volume of 10 mL/kg and a respiratory rate of 60 breaths/min with a positive end-expiratory pressure (PEEP) set at 3 cmH_2_O. Following laparotomy and sternotomy we injected 300 IU heparin into the inferior vena cava and sacrificed the rats by clamping the ascending aorta. The pulmonary artery and the left atrium perfusion cannulae (Hugo Sachs, Hugstetten, Germany) were then inserted. We set a perfusion pressure of 20 cmH_2_O and flushed both lungs with 20 mL of cold low-potassium dextran solution (Perfadex plus, Xvivo Perfusion, Göteborg, Sweden) through the pulmonary artery cannula. The Perfadex plus solution was designated for the storage of lung transplants. At 4°C, and out from a sealed Perfadex plus bag the pH was of 7.4 and the oxygen tension (pO2) was of 35.19 kPa. Perfadex plus contain 50 g/l of Dextran 40, 6 mmol/l potassium, 138 mmol/l sodium, 0.8 mmol/l magnesium, 132 mmol/l chloride, 0.8 mmol/l sulphates, 0.8 mmol/l phosphates, 0.5 mmol/l calcium chloride, 5 mmol/l glucose, 0.24 ml/l THAM-buffer and has 325 mOsm/l of osmolarity. The deflated lungs were left in the rat, *in situ* [17], and at room temperature (20°C) for 2 hours of warm ischemic time. After the 2 hours, the heart lung block were weighted and then perfused for 4 hours in an Isolated Perfused Lung system for rat and guinea pig system (IPL-2, Hugo Sachs Elektronik Harvard Apparatus, Germany) at different subnormothermic perfusion temperatures of 21°C, 24°C, 28°C and 32°C as well as a standard normothermic temperature of 37°C. At the end of the 4 hours of EVLP, we performed an additional 5 minutes stress test where the perfusate flow was enabled to increase until automatized IPL-2 controller system reached a maximum pulmonary arterial pressure (PAP) of 15 cmH_2_O. The mean flow value with standard deviation recorded in the different groups were the following: a) 8.475 +/- 2.032 ml/min for the 21°C group, b) 15.65 +/- 3.156 ml/min for the 24°C group, c) 15.68 +/- 3.519 ml/min for the 28°C group, d) 16.65 +/- 1.428 ml/min for the 32°C group and d) 14.63 +/- 2.503 ml/min for the 37°C group.

#### Physiological variables and clinical biochemistry parameters

We monitored all the respiratory parameters under positive pressure ventilation with a dedicated software (PULMODYN® software, Hugo Sachs Elektronik Harvard Apparatus, Germany) for 4 hours and recorded continuously the pulmonary artery pressure, the peak airway pressure, the airway flow, the dynamic lung compliance (Cdyn) and the pulmonary vascular resistance (PVR).

Hourly, and 5 min after switching lung ventilation with a fraction of inspired oxygen (FiO_2_) of 1, we collected samples for pH, partial pressure of oxygen (PaO_2_), concentration of potassium, calcium, sodium, glucose, chlorine and lactate measurement with the Epoc® blood analysis system (Epoc® Blood Analysis System, Siemens Healthineers, Erlangen, Germany). The change in PaO_2_ (ΔPaO_2_) was calculated as partial pressure of the pulmonary venous PO_2_ –pulmonary arterial PO_2_.

#### Normothermic and subnormothermic EVLP treatment groups

As an acellular perfusate, we selected a starting volume of 125 mL of Steen solution (Steen solution, XVIVO Perfusion AB, Göteborg, Sweden) supplemented with 300 IU sodium heparin, antibiotic (50 mg meropenem), and methylprednisolone (50 mg Solu-Medrol, Pfizer Inc., New York, USA) that was recirculated during the 4 hours of EVLP (about 23 times recirculated). Before the start of the EVLP procedure, we oxygenated the Steen perfusate solution. The equilibrium of perfusate’s dissolved O_2_ content was reached at 20°C after 15 min at a 30 mL/min flow through a gas exchange membrane (D-150 hemofilter, Medsulfone, Italy) with 2 liters per minute flow of 100% oxygen. The 100% targeted flow was calculated as the 20% of a 250 g weight rat with a 75 mL/min cardiac output. We started lung perfusion with 10% of the targeted flow (1.5 mL/min) for 10 minutes. The five following 10 minutes steps in ml/min were 3 ml/min, 4.5 ml/min, 7.5 ml/min, 12 ml/min (120 mL). Then at the 50 min time point we switched to maximum flow of 15 ml/min and kept it for 3 hours and 10 minute until the end of the experiment. The left atrium pressure was set at 2–3 cmH_2_O and the automatized IPL-2 controller system maintained the pulmonary arterial pressure (PAP) below 15 cmH_2_O by adjusting the flow. The circuit perfusate temperature was set at 20°C to match the room temperature of 20°C used during the 2 hours warm ischemic time and was gradually increased using a thermostatic water bath and the targeted temperatures were reached after 15 minutes for the of 21°C, 24°C and 28°C groups and after 25 minutes for the 32°C and 37°C groups and kept onwards during the 4-hour time of EVLP. Ventilation with the IPL-2 ventilator (VCM-P, Hugo Sachs Elektronik Harvard Apparatus, Germany) started for all the groups after 20 min reperfusion time with a fixed tidal volume of 5 mL/kg, an inspiratory/expiratory ratio of 1/3 and a rate of 30 breaths/min and with a PEEP of 3 cmH_2_O and a FiO_2_ of 0.21. Thereafter, the perfusate was constantly deoxygenated with a mixture of 8% CO_2_ and 92% N_2_ using a gas exchange membrane (D-150 hemofilter, Medsulfone, Italy).

### Biochemical measurements

#### Cytokines, chemokines and mediators of tissue repair

The perfusate collected after 4 hours of EVLP were flash frozen in liquid nitrogen and stored at –80°C until testing with a rat multiplex panel (Milliplex® Magnetic Bead Panel; Millipore, Billerica, MA, USA) according to the manufacturer’s instructions. We assessed 50 μL of the perfusate for different interleukins (IL) IL-1α, IL-1β, IL-6, IL-18, and chemokines such as monocyte chemoattractant protein 1 (MCP-1), macrophage inflammatory protein-2 (MIP2), macrophage inflammatory protein-1α (MIP-1α) GRO/KC/CINC-1 and for the growth factor modulator of tissue repair VEGF.

#### ATP content, myeloperoxidase activity, carbonylated protein assays in lung tissues

At the end of EVLP, the rat lung tissues were collected and flash frozen in liquid nitrogen and then stored at -80°C. Frozen lung tissues were crushed on dry ice and the powdered tissues were used to prepare the lysates. ATP content was determined from 25 mg of powdered lung tissue and extracted in 0.5 mL of 0.5% trichloroacetic acid and centrifuged at 8000 rpm for 2 min at 4°C. The supernatant was isolated, and 10X Tris-acetate was added to neutralize the pH to 7.4 with a 10 μL of 0.002% xylenol blue used as pH indicator. ATP concentration in the supernatant was determined enzymatically with an ATP assay kit (Enliten,; Promega, Madison, WI, USA) by measuring in the luminescence channel of a Cytation 5 plate reader (BioTek Instruments, Inc., Winooski, VT, USA). Tissue lysates extracted from the powdered lung tissue were also analyzed using 1) a myeloperoxidase (MPO) activity assay (OxiSelect™ myeloperoxidase chlorination activity assay, Cell Biolabs San Diego, CA, USA) and 2) an ELISA-based carbonylated proteins assay (OxiSelect™ carbonyl protein ELISA assay, Cell Biolabs San Diego, CA, USA) according to manufacturer’s instructions. A microBCA protein assay kit was used with bovine serum albumin as standard to determine protein concentration in the supernatant according to the manufacturer’s instructions (Thermo Scientific, Rockford, Il, USA).

### Lung tissue TUNEL analysis after EVLP

A terminal deoxynucleotidyl transferase dUTP nick end labeling (TUNEL) assay was performed on Leica Bond RX using Refine AP-Kit (Leica DS9800) including all the buffer-solutions from Leica Microsystems Newcastle, Ltd. and processed according to the manufacturer guidelines. The antigen retrieval was performed with LEICA Enzyme Pretreatment Kit Cat. AR9551 for 25 min at 37°C. Paraffin-slides were dewaxed, pretreated and incubated with mouse anti-DIG FITC Jackson Immuno-Research, Cat: 200-092-156 at 1:500 dilution and a rabbit anti-FITC (Serotec Cat. 4510–7804 at 1:1000 dilution + 2% normal mouse serum). The TdT-Enzyme were the following; 1 mL antibody-diluent (Leica AR9352), 100 μL TdT-Buffer ((TUNEL-Box), M1893, Promega, Madison, WI, USA), 1 μL dUTP (TUNEL-Box) DIG-11-dUTP (Roche Cat: 11 570013 910), 4 μL TdT-Enzyme ((TUNEL-Box), M1875, Promega, Madison, WI, USA). The cytoplasm of apoptotic cells contained red granules and the number of apoptotic cells in 10 random high-power fields (X400) was calculated. The apoptotic index was expressed as the number of apoptotic cells/100 cells (%).

### Statistical method

Results are expressed as mean and standard deviation (SD). For cytokine analysis the median and interquartile range *(*IQR*)* was used as measures of central tendency and dispersion, respectively. A nonparametric Mann–Whitney U-test was used for non-continuous data. Data with a time component were compared without assuming sphericity using 2-way analysis of variance (ANOVA) and the Geisser-Greenhouse correction. Statistical analysis was performed with GraphPad Prism version 8 software (GraphPad Software, Inc., La Jolla, CA, USA). Differences were considered significant at *p<0*.*05*.

## Results

### Lung physiology during EVLP

**Fig 1:**
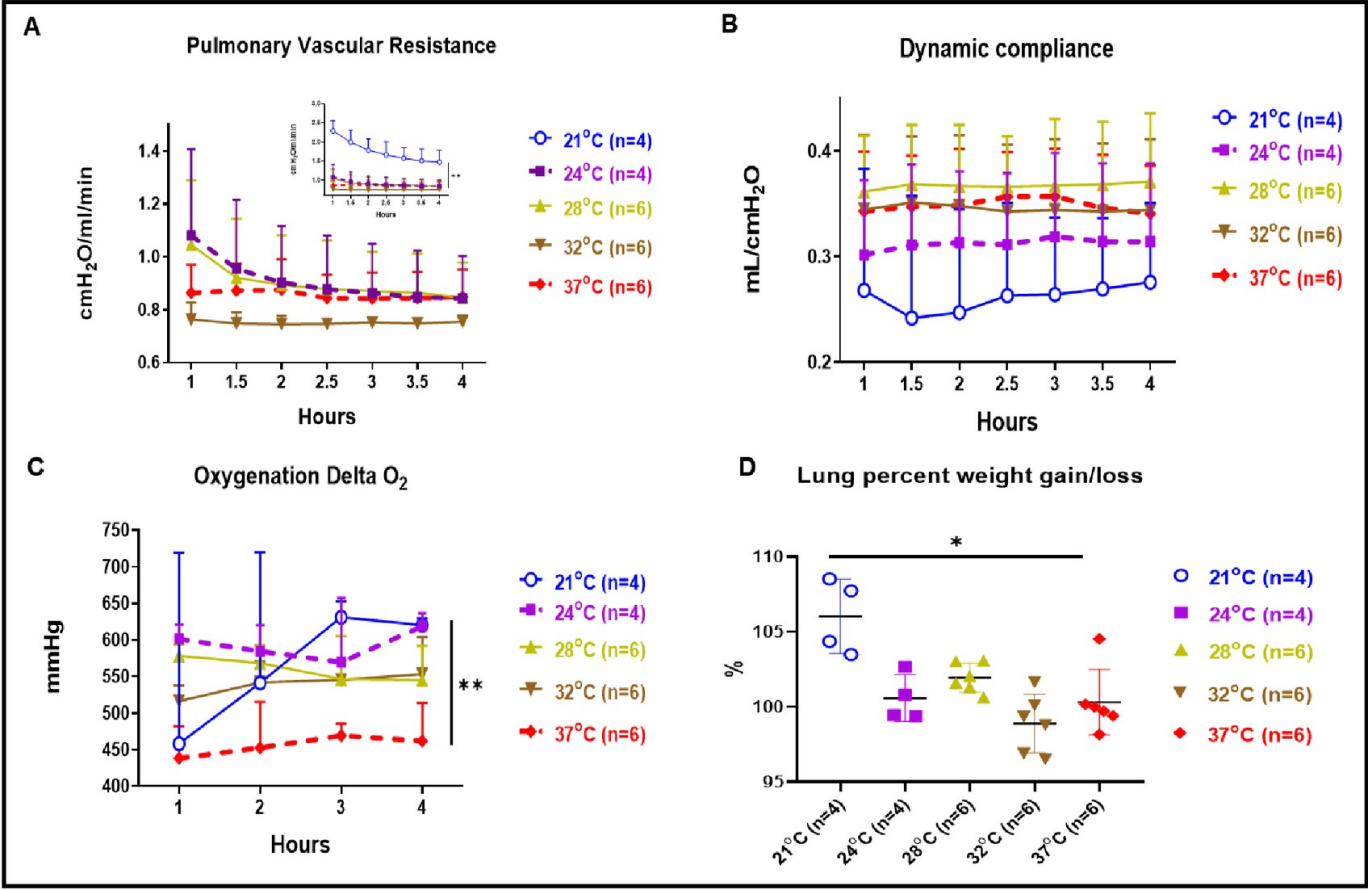
Physiological variables and oxygenation parameters. (A) PVR at 21°C was significantly higher than those at 37°C (*p<0*.*01*) (see Fig 1A insert). PVR at 32°C were with a lower but not statistically different value compared to PVR at 37°C. PVR at 28°C and 24°C were comparable to PVR at 37°C. (B) Cdyn at 21°C and 24°C showed a not significantly different lower value than those at 37°C. Cdyn at 28°C and 32°C were similar to Cdyn at 37°C. (C) Perfusate oxygenation (delta PO_2_ or left atrial pressure of oxygen minus pulmonary arterial pressure of oxygen) was significantly improved at 24°C and 21°C (*p<0*.*01*) and were higher at 28°C and 32°C although this difference was not statistically different from the control EVLP performed at 37°C. (D) Percentage of gain/loss in lung weight after 4 hours of EVLP was significantly higher at 21°C when compared to 37°C normothermic temperature (*p<0*.*05*).

PVR at 21°C showed significantly higher values than all the other perfusion temperature conditions (Fig 1A insert, *p<0*.*01*). We observed that the PVR within the 4 hours at 32°C subnormothermic was also lower but this difference was not statistically different from the control EVLP performed at 37°C. The PVR value at 4 hours of EVLP done at 24°C and 28°C were comparable to the PVR values at 37°C. Cdyn at 28°C showed higher values toward that recorded at 37°C, but was not statistically different (Fig 1B) whereas Cdyn at 21°C and 24°C showed a not statistically different lower values. Cdyn values in EVLP done at 32°C and 28°C were overlapping the one recorded at 37°C. The oxygenation at subnormothermic 21°C and 24°C temperatures showed a statistically better oxygenation compared to that of 37°C (Fig 1C, *p<0*.*01*) whereas oxygenation at 28°C and 32°C were also higher although this difference was not statistically different from the control EVLP performed at 37°C. We also observed edema formation after 4 hours of EVLP for the subnormothermic 21°C condition with a significantly higher percentage gain of lung weight when compared to 4 hours of EVLP performed at 37°C (Fig 1D, *p<0*.*05*).

### Clinical biochemistry parameters

**Fig 2:**
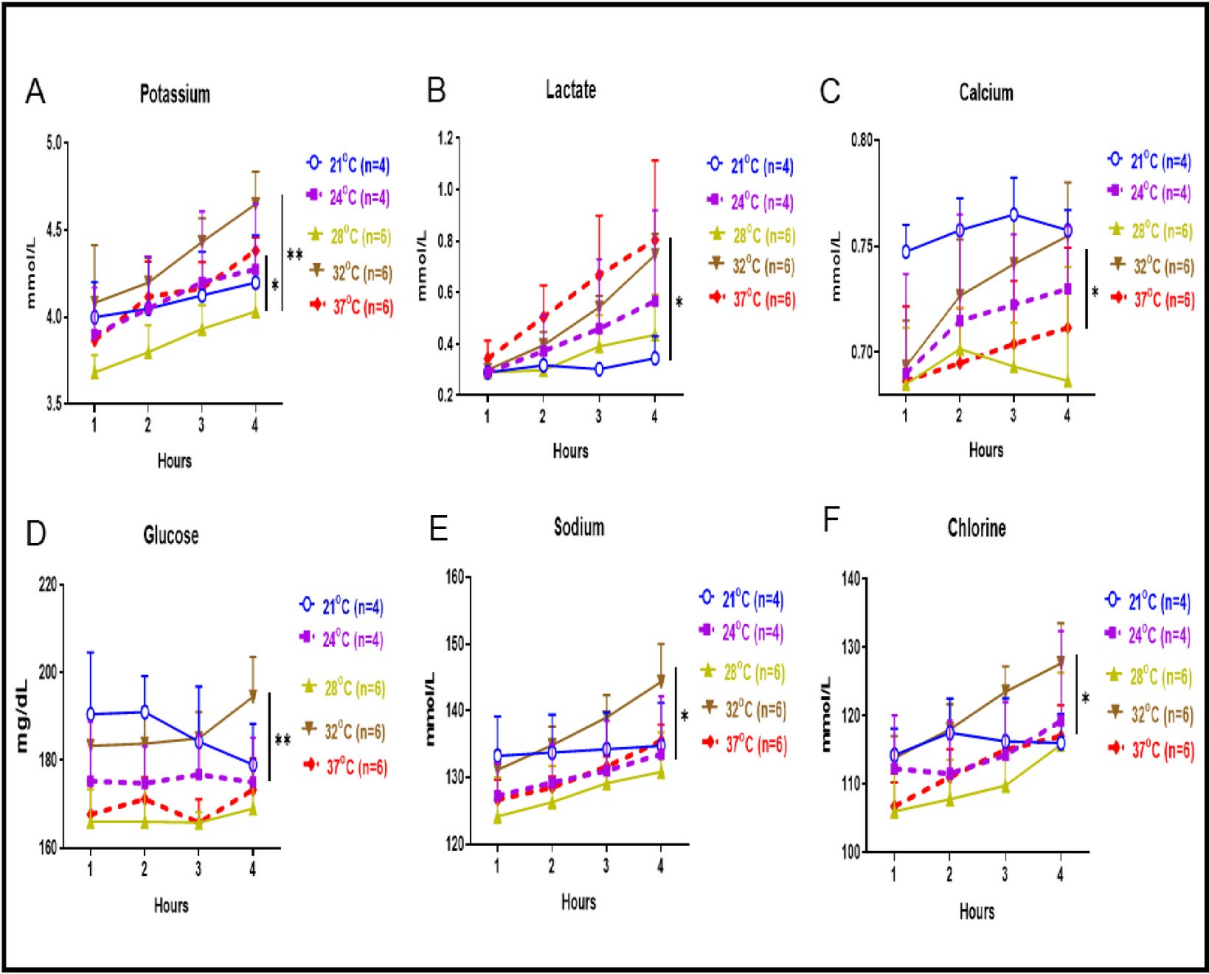
Clinical biochemistry parameters. (A) Concentration of potassium at 28°C was significantly lower than 37°C and 32°C (*p<0*.*05 and p<0*.*01 respectively*), but potassium ion concentration obtained at 24°C and 21°C was similar to that of 37°C. (B) Lactate levels at 21°C and 28°C were significantly lower than that of 37°C (*p<0*.*05*). Lactate levels at 32°C and 24°C were similar to that of 37°C. (C) Calcium ion perfusate concentrations was at their lowest level at 28°C, higher at 32°C and 24°C and were significantly higher at 21°C compared to the 37°C condition (*p<0*.*05*). (D) Perfusate glucose concentration was significantly higher at 32°C compared to the 37°C condition (*p<0*.*01*). (E) Sodium ion concentration was at their lowest level at 28°C and significantly higher at 32°C compared to the normothermic 37°C (*p<0*.*05*). (F) Chlorine ion concentrations was at their lowest level at 28°C and significantly higher at 32°C compared to the normothermic 37°C (*p<0*.*05*).

Perfusate concentrations of potassium ions at 28°C were significantly lower than that of 37°C and 32°C (Fig 2A, *p<0*.*05 and p<0*.*01*, respectively). Concentration for potassium ions at 24°C and 21°C were similar to that of 37°C. Perfusate concentrations of lactate at 21°C and 28°C were significantly lower than that of 37°C (Fig 2B, *p<0*.*05*). Calcium concentrations after 4 hours were at their lowest level at 28°C, higher at 32°C and 24°C and significantly higher at 21°C compared to the normothermic 37°C condition, (Fig 2C, *p<0*.*05*). Perfusate glucose concentrations were significantly higher at 32°C compared to the normothermic 37°C condition, (Fig 2D, *p<0*.*01*). Concentrations of sodium and chlorine were at their lowest level at 28°C whereas at 32°C they were significantly higher than that of 37°C (Fig 2E and 2F *p<0*.*05* respectively).

### Perfusate cytokines, chemokines and mediators of tissue repair

**Fig 3:**
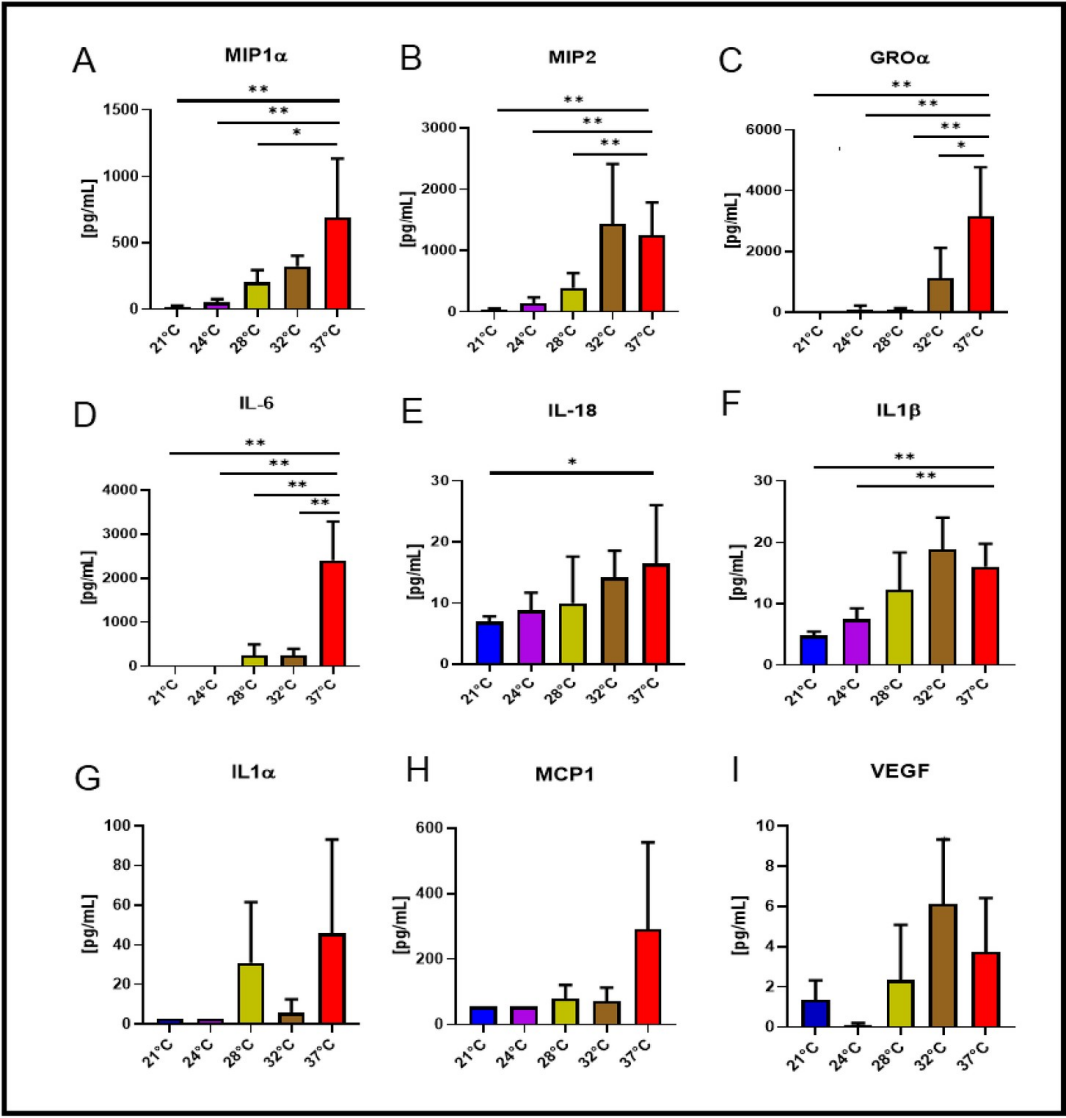
Cytokines and chemokines in the perfusate. After 4 hours of EVLP, concentrations of chemokines MIP1α (Fig 3A) and MIP2 (Fig 3B) collected from the 28°C, 24°C and 21°C conditions were significantly lower than at 37°C (*p<0*.*05 to p<0*.*01*). Concentrations of GRO-α (Fig 3C) and IL-6 (Fig 3D) collected from all the subnormothermic conditions were significantly lower than at 37°C (*p<0*.*05 to p<0*.*01*). Concentration of IL-18 (Fig 3E) at 21°C was significantly lower than at 37°C (*p<0*.*05*), whereas IL-18 levels detected at 24°C, 28°C and 32°C were lower but not significantly different from levels observed at 37°C. Concentrations of IL-1β (Fig 3F) at 21°C and 24°C were lower than at 37°C (*p<0*.*01*). For IL-1α (Fig 3G), MCP-1 (Fig 3H) and VEGF (Fig 3I), their concentrations showed lower values compared to 37°C.

Both macrophage inflammatory protein-1α (MIP-1α) and macrophage inflammatory protein-2 (MIP2) concentrations at 21°C, 24°C and 28°C were significantly lower than at 37°C (Fig 3A and 3B, *p<0*.*05 to p<0*.*01*), but that of 32°C were not statistically different from the normothermic condition. Growth-regulated oncogene-α (GRO-α) and interleukin-6 (IL-6) concentrations were significantly lower at all subnormothermic temperatures comparing to 37°C (Fig 3C and 3D, *p<0*.*05 to p<0*.*01*). Interleukin-18 (IL-18) levels at 21°C were significantly lower than the levels of the normothermic condition (Fig 3E, *p<0*.*05*) whereas IL-18 levels detected at 24°C, 28°C and 32°C showed lower but not statistically different values toward levels observed at 37°C. Interleukin-1β (IL-1β) levels at 21°C and 24°C were statistically lower than levels of IL-1β at 37°C (Fig 3F, *p<0*.*01*). For Interleukin-1α (IL-1α, Fig 3G), Monocyte chemoattractant protein 1 (MCP-1, Fig 3H) and Vascular endothelial growth factor (VEGF, Fig 3I), concentrations showed only reduced concentration values but not statistically different compared to 37°C concentrations.

### TUNEL assay, myeloperoxidase activity, ATP and carbonylated protein content in lung tissues

**Fig 4:**
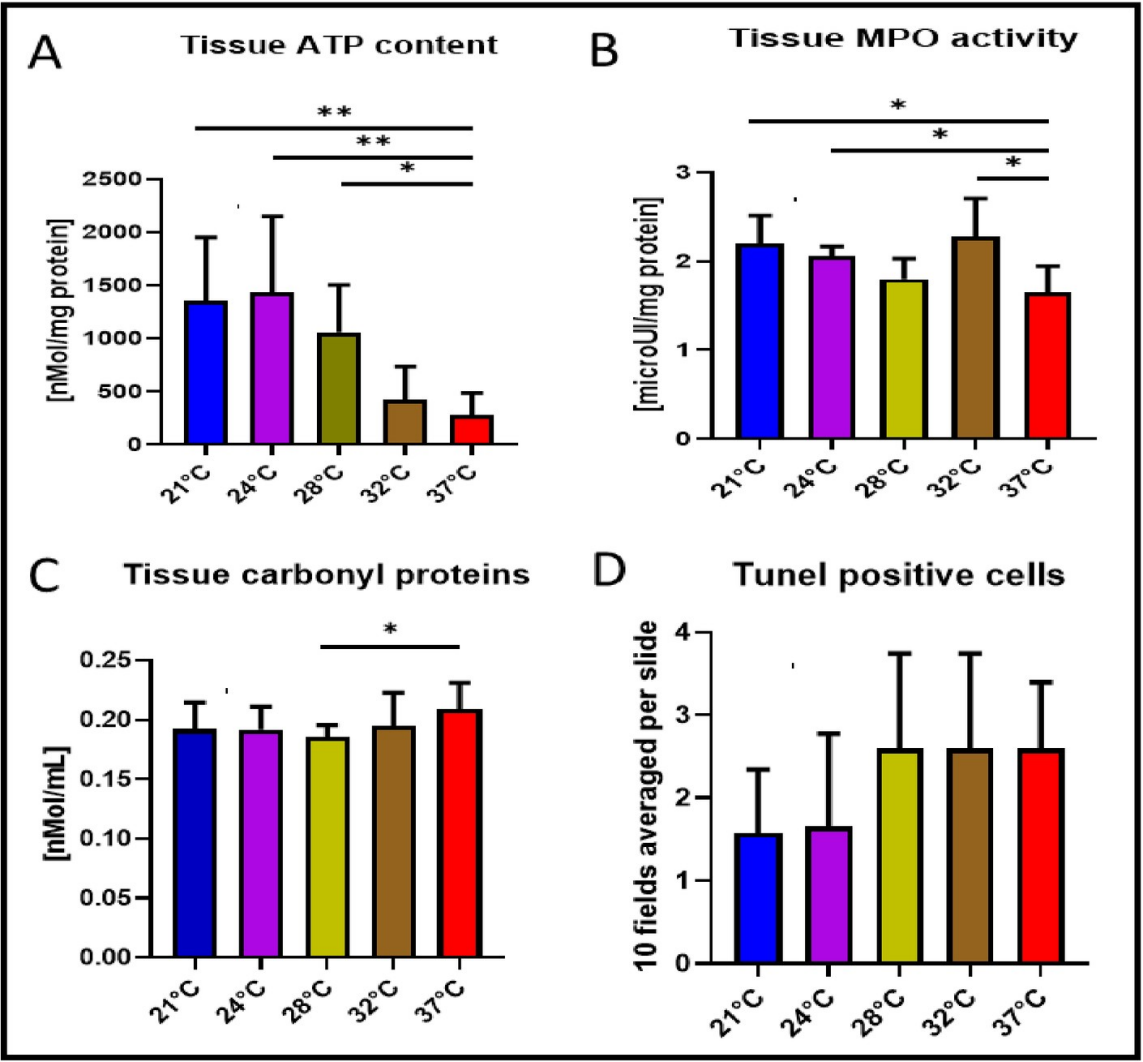
TUNEL assay, Myeloperoxidase activity, ATP and carbonylated protein content in lung tissues. (A) ATP content in lung tissue was significantly higher at 24°C and 21°C (*p<*0.01) or 28°C (*p<0*.*05*) versus ATP tissue content at 37°C. (B) MPO activity in lung tissue after 4 hours of EVLP at 28°C were close to the value at 37°C, whereas significantly higher MPO activity were observed in tissue from EVLP performed at 21°C or 24°C and 32°C (*p<0*.*05*). (C) Carbonylated protein level was significantly lower at 28°C (*p<0*.*05*) versus lung tissue exposed to 37°C EVLP. (D) TUNEL analysis in histological preparation of lung tissue showed lower but not statistically different values towards less apoptotic cell damage in subnormothermic temperature of 24°C and 21°C.

In Fig 4A, the tissue ATP content at 28°C (*p<0*.*05*) and 21°C (*p<0*.*01*) or 24°C (*p<0*.*01*) were significantly higher than at 37°C. ATP content of tissue with EVLP performed at 32°C was comparable to normothermic condition. As shown in Fig 4B, the MPO activities detected in lung tissue from the 28°C and 37°C EVLP were similar whereas significantly higher MPO activities were observed in tissue from EVLP performed at 21°C, 24°C and 32°C (*p<0*.*05*). Carbonylated protein contents in lung tissues after 28°C EVLP were significantly lower than that of 37°C (Fig 4C, *p<0*.*05*) but there was no statistical differences among 21°C, 24°C and 32°C conditions compared to the normothermic temperature. TUNEL analysis in lung tissue showed lower but not statistically different values towards less apoptotic cell damage in subnormothermic temperature of 24°C and 21°C (Fig 4D).

## Discussion

Since the implementation of DCD lung transplantation in some countries, this procedure has dramatically increased the donation rate by 20 to 50% [18]. The advent of EVLP in lung transplantation has also shown immense potential, which has resulted in improved post-transplant outcomes. The DCD lung physiology is degraded due to the metabolic damages sustained by the warm ischemic time [19]. Nonetheless in 2001, the group of Steen el al [20] restored with EVLP the oxygenation and the substrates flow to human DCD lungs and performed the first successful human lung transplantation of uncontrolled DCD donors. Normothermic perfusion temperature is the current standard for machine perfusion assessment of the lung before transplantation. However, subnormothermic perfusion temperature settings are already in human clinical use among several human solid organs or tested in animals [6–11, 14, 15]. In order to evaluate if subnormothermic temperatures might impact the physiological, biochemical and immunological parameters of the lungs, we exposed rat lungs for 2 hours of warm ischemic time at room temperature and then performed subnormothermic machine perfusion for 4 hours at 32°C, 28°C, 24°C and 21°C with a dedicated rat EVLP platform. We found that EVLP at 28°C compared to the normorthermic situation had acceptable physiological variables, significant improvement in tissue ATP content and both decreased tissue MPO activity and carbonylated proteins with reduced release of pro-inflammatory cytokines.

The use of acellular machine perfusion for organ preservation [21] and the application of hypothermia was already described in liver [9, 10, 22–24] in heart [25, 26] in kidney [11] in injured lungs [27], and in rat lung machine perfusion [12, 13, 28, 29]. The assessment of donor lung quality during EVLP mainly relies on the monitoring of airway pressure, pulmonary artery pressure, and oxygen concentration. Here, in a rat EVLP model of DCD lung donors we report that the oxygenation data were significantly improved at 21°C and 24°C but not statistically different than those at 32°C and 28°C. An obvious physicochemical explanation for this improved oxygenation data is that gases are better kept in a soluble phase with low temperature acellular perfusate as compared to perfusion done at 37°C.

The PVR value of EVLP done at 32°C showed a not statistically different lower values compared to the 37°C EVLP condition whereas the Cdyn values at 28°C and 32°C were comparable to the 37°C. An hypothesis for the poor PVR and Cdyn values at 21°C and 24°C may have been the deleterious effect of cold on either the blood vessels (vasoconstriction) or the lung parenchyma or on the biophysical properties of surfactant. As a consequence of this, we observed a significant higher weight for the lung perfused at 21°C due to edema formation when compared to normothermic temperature. It has been reported that during the months of hibernation which is a natural and complex physiological and metabolic response to cold temperature developed in mammals that lung present tissue remodelling [30–32] and that the lung surfactant composition was also adapted to the cold [33]. In comparison to those long cold hibernating time adaptation of the lung in our EVLP setting was short and did probably not allow enough time for any lung tissue remodelling. In brief, all these physiological data (PVR, edema, Cdyn, delta PO_2_,) point towards a protective effect of subnormothermic perfusion temperature in the 28°C to 32°C range with a better physiological state of the DCD donor graft after 4 hours of EVLP compared to the 37°C situation. Contrary to this, we observed a more deleterious effect of subnormothermic temperature on the Cdyn and edema formation of the lungs treated at 24°C and 21°C.

As both the liver and kidneys are absent from acellular organ machine perfusion protocols, the circulating concentration of potassium, sodium, and lactate increases as the pH drops. We recorded comparable values for pH (data not shown), sodium and chlorine at 28°C when compared to 37°C situation. The potassium levels in a perfusate may also be used as an indirect proxy for cell integrity [34] and cell death [35]. We obtained at 28°C significantly lower concentrations of potassium versus the 37°C and 32°C conditions, whereas sodium and chlorine concentrations had a lower but not significantly different values at 28°C versus the normothermic condition. The low potassium levels might be explained by less cell lysis at low perfusion temperature. The anaerobic glycolysis is the process by which the normal pathway of glycolysis is routed to produce lactate and this metabolic waste products will accumulate over time [36]. Lactate accumulation occurs at times when energy is required in the absence of oxygen. The preoperative serum lactate levels is a strong independent predictor of worse outcomes in patients undergoing urgent heart transplant on short-term mechanical circulatory support [37]. Nonetheless, for patients who underwent lung transplantation after EVLP had good outcomes despite high lactate during EVLP [36]. In our experiments, the lactate concentrations at 28°C and at 21°C were significantly lower than those at 37°C and we could also record a gradual decrease/consumption of glucose in all of the subnormothermic or control perfusate conditions but the consumption of glucose were only significantly higher at 32°C. Since there are no compensatory mechanisms in this EVLP setting, the decreased lactate levels or reduced glucose consumption levels may indicate respectively reduced anaerobic glycolysis in the 28°C and 21°C groups and higher anaerobic glycolysis in the 32°C group. These changes may mainly be attributable to the pneumocyte metabolism that occurs when energy is required in the absence of oxygen. When compared to the normothermic temperature, the concentrations of calcium were significantly higher at 21°C. This hypercalcemia is possibly related to the cell death related release of intracellular calcium into the perfusate and this excessive entry of calcium into the lung parenchyma might have damaged it or even caused it to undergo apoptosis, or death by necrosis. Calcium also acts as one of the primary regulators of osmotic stress and we observed a significant higher weight of the lungs after EVLP done at 21°C. All these clinical chemistry data in EVLP perfusate for potassium, calcium, sodium and chlorine ions but also lactate and glucose point towards a beneficial effect of the 28°C subnormothermic perfusate temperature compared to the 37°C situation and a rather deleterious effect at 21°C and 32°C temperature.

We selected a panel of cytokines, interleukins and growth factors to monitor their release during EVLP-induced I/R injury. Some pro-inflammatory cytokines and chemokines accumulate during EVLP and correlate with higher rates of primary graft dysfunction of the transplanted lungs [38]. In the perfusate at 28°C, the detected amount of chemokines MIP1α, MIP2, GRO-α (KC is a rat homolog of human GRO-α) and the cytokine IL-6 were significantly lower than at 37°C. Other proteins recovered such as the chemokine MCP1, the cytokines IL-18, IL1β and IL1α were not always detected in each perfusates but they nonetheless showed a lower but not significantly different release at subnormothermic temperature ranging from 21°C to 32°C when compared to the 37°C EVLP condition. We also observed the same gradual but not statistically different reduction of VEGF collected at subnormothermic temperature of 21°C, 24°C and 28°C when compared to the value at 37°C.

Finally, several biochemical parameters were also improved with sunormothermic perfusion temperature. Generally, I/R injury occurs when the blood supply to tissue is blocked (ischemic time) [39]. It has been hypothesized that after stopping the flow in the lung endothelium and during the cold preservation graft ischemic time but also when flow is reinstated (reperfusion time) that a mechanosignaling cascade initiate a sodium ions influx and persistent cell membrane depolarization due to a decrease of intracellular potassium ions. Moreover, during the graft cold ischemic time the cell calcium and ATP storage are depleted leading to alteration in mitochondrial membrane permeability and depolarization of their membrane potential [40]. Once organ reperfusion is performed the mitochondrial permeability transition triggers both mitochondrial reactive oxygen species, generation and opening of mitochondrial permeability transition pore, ultimately leading to damage-associated molecular patterns release and tissue inflammation [41]. The ATP tissue content at the end of EVLP was significantly higher at 24°C, 21°C and 28°C showing an increased protective effect of these subnormothermic temperatures on keeping optimal mitochondrial activity. The reactive oxygen species are known to be a factor of injury during hypothermic time [42] both in liver [22, 43] and lung [44]. Protein carbonylation is a type of protein oxidation that can be promoted by reactive oxygen species [45, 46]. The tissue content in carbonylated proteins were significantly lower at 28°C whereas only a not significantly different decrease of TUNEL quantified apoptotic cells could be observed in subnormothermic temperature of 24°C and 21°C. Moreover, MPO enzymes which are released into the extracellular fluid as a result of oxidative stress or during an inflammatory response and probably issued from neutrophils, were significantly more active under 21°C, 24°C and 32°C subnormothermic conditions but MPO activities at 28°C remained close to the value at 37°C. All these biochemical parameters in lung tissue after 4 hours of EVLP (ATP, apoptotic cells content, MPO and carbonyl proteins) point towards a beneficial effect of the 28°C temperature in the biochemical parameters of the DCD donor graft after 4 hours of EVLP compared to the 37°C situation and a rather deleterious effect of the 32°C, 24°C and 21°C temperatures.

Our study had some limitations. Lung function was assessed only during EVLP, but no further lung transplantation was performed and we did not use a leucocyte filter during EVLP which was documented to reduce the release of pro-inflammatory cytokine IL-6 in the perfusate and which absence impair the quality of the lung grafts [47].

Among our subnormothermic conditions, the 28°C perfusion was found to be the most promising because of acceptable physiology and both reduced energy consumption and immune response. EVLP with perfusate temperature of 28°C were physiologically comparable to the 37°C whereas EVLP at 21°C showed the worst lung physiological data. Nevertheless, at all the EVLP temperature lower than 28°C, low energy consumption and low inflammatory response were recorded. Recently, Gloria et al. presented evidences after a 2-hour reperfusion time in transplanted recipient rats, that lung grafts treated pre-transplantation without EVLP but exposed to a 4-hour cold ischemic time have a worst outcome than their three experimental groups of undamaged lungs pretreated before transplantation with EVLP temperature set at either 25°C, 30°C or 37°C [14]. We recently published a study [15] where all the rat HBD lungs were pre-damaged by exposure to a 1-hour cold ischemic time to mimic as close as possible a clinical situation. In this study, and compared to the control 37°C normothermic EVLP setting, the HBD damaged lungs showed improved physiological parameters and attenuated I/R injuries after both the 28°C subnormothermic for 4-hour EVLP time alone or in a combination of the 28°C subnormothermic for 4-hour EVLP time followed by a 2-hour time with left lung transplantation. The present article is the first study directly comparing different subnormothermic EVLP temperature with DCD rats donor lungs and we show that perfusion at 28°C have a strong benefit potential for both physiological parameters and attenuation of I/R injury. Taken together all of our data, this study suggests that 28°C EVLP is a non inferior setting in comparison to the clinically approved 37°C EVLP and may potentially reduce lung I/R injury of DCD donors. Large animal models are needed to further prove this conclusion.

## Abbreviations

Cdyn: Dynamic lung compliance
DCD: Donation after circulatory death
EVLP: *Ex vivo* lung perfusion
FiO_2_: Fraction of inspired oxygen
I/R: Ischemia-reperfusion
PAP: Pulmonary arterial pressure
PEEP: Positive end-expiratory pressure
MPO: Myeloperoxidase
PVR: Pulmonary vascular resistance
TUNEL: Terminal deoxynucleotidyl transferase dUTP nick end labeling
